# Coronary Stent Fracture Causing Myocardial Infarction: Case Report and Review of Literature

**DOI:** 10.31083/j.rcm2311384

**Published:** 2022-11-16

**Authors:** Matthias Gröger, Wolfgang Rottbauer, Mirjam Keßler

**Affiliations:** ^1^Department of Internal Medicine II, University of Ulm, 89081 Ulm, Germany

**Keywords:** stent fracture, coronary stent fracture, acute coronary syndrome

## Abstract

Coronary stent fracture (SF) is a potential cause of stent failure increasing the risk for in-stent restenosis, stent thrombosis, target lesion revascularization and major adverse cardiac events. Overall incidence of SF ranges from <1.0% up to 18.6% and SF can be found in up to 60% of failed devices. Advanced imaging techniques have improved the detection of SF. However, defining the optimal therapeutic approach towards these complex lesions is challenging. This review summarizes the most important publications on the topic of SF and discusses current insights into pathophysiology, diagnostic tools, classification and therapeutic management. Furthermore, two illustrative cases of SF leading to myocardial infarction are presented, which demonstrate typical SF risk factors such as vessel angulation and hinge motion, stenting in the right coronary artery, use of long stents and multiple stent layers.

## 1. Introduction

The evolution of percutaneous coronary intervention (PCI) to treat coronary stenosis and acute coronary syndrome is a success story. Stent technology has greatly improved since the first implantation of a stainless steel wire-mesh stent in a human coronary artery by Sigwart and Puel in 1986 [1]. Ground-breaking bio-mechanical advances have led to the development of first generation bare metal stents (BMS) and later drug-eluting stents (DES). While BMS addressed the issues of flow-limiting dissection, recoil and restenosis, the broad use of DES further decreased rates of in-stent restenosis (ISR), stent thrombosis and target lesion revascularization (TLR) [2]. However, stent failure including stent fracture has remained a hazard for patients and a potential challenge for interventional cardiologists. Initially being thought to have a generally benign clinical course, SF has been linked to adverse results such as stent failure and major adverse cardiac events (MACE) and has therefore raised increasing awareness among interventionalists [3, 4]. In this review, we describe two illustrating cases of stent fractures followed by an analysis of current literature on the pathomechanism, diagnostic tools, classification and therapeutic management of coronary stent fractures.

## 2. Methods

We performed a systematic literature research using the scientific databases PubMed and Cochrane. Search terms were “stent fracture”, “coronary stent fracture”, “drug-eluting stent fracture”, “DES fracture”, “stent thrombosis”, “DES thrombosis”.

## 3. Case Report

**Fig. 1.**
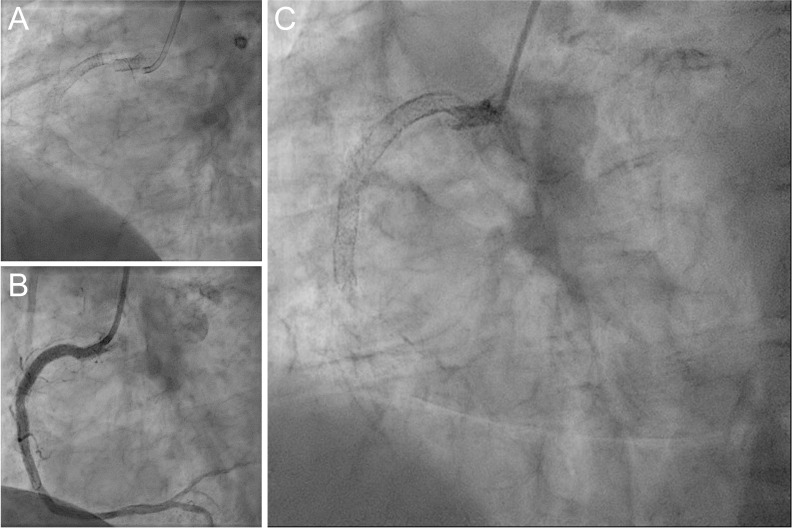
**Coronary angiogram of the right coronary artery of patient 1**. Initial complete SF of a Xience EES, that had lead to STEMI (A), was initially treated with deployment of 1 Promus PREMIER DES and later stabilized by a second EES and 1 Onyx ZES (B). However, recurrent type-IV-SF with TIMI-0-flow was observed in a setting of NSTEMI (C).

Patient 1, a 62-year-old male, had undergone percutaneous coronary intervention (PCI) of a high-grade stenosis of the proximal right coronary artery (RCA) with implantation of one everolimus-eluting stent (EES) (4.0 × 15 mm, Xience Xpedition, Abbott Vascular, Santa Clara, CA, USA). Recurrent ISR of the proximal RCA had led to Re-PCI with implantation of one sirolimus-eluting stent (SES) (4.0 × 30 mm, Orsiro, Biotronik AG, Berlin, Germany) and one EES (4.0 × 28 mm, Xience V, Abbott Vascular, Santa Clara, CA, USA). Afterwards the patient had been asymptomatic. After two years, the patient had undergone another PCI on admission due to inferior ST-Segment-Elevation myocardial infarction (STEMI). Emergent coronary angiography (CAG) had identified a gap within the previously implanted DES in the proximal RCA, indicative of a complete stent fracture (type IV) (Fig. 1A). Discontinuous TIMI-II-flow had been observed in the RCA. One platinum-chromium alloyed EES stent (3.5 × 20 mm, Promus PREMIER, Boston Scientific, Marlborough, MA, USA) had been deployed. ECG abnormalities had eventually returned to normal and the patient had turned asymptomatic.

Short-term follow-up CAG had shown persistent excessive motion at the hinge point of the RCA within the Promus PREMIER EES indicating strong mechanical strain. At the initial SF site of the RCA, one Onyx zotarolimus-eluting stent (ZES) (4.0 × 22 mm, Medtronic, Minneapolis, MN, USA) had been deployed to further stabilize the Promus PREMIER EES. Final CAG had shown an optimal final result of the RCA (Fig. 1B).

At the latest presentation, 4 years after the initial PCI, the patient was again referred to CAG due to Non-ST-Segment-Elevation myocardial infarction (NSTEMI). The CAG again showed SF of all deployed stent layers in the proximal RCA (Fig. 1C). Further coronary intervention was deferred and the patient was transferred to cardiac surgery for single bypass surgery.

**Fig. 2.**
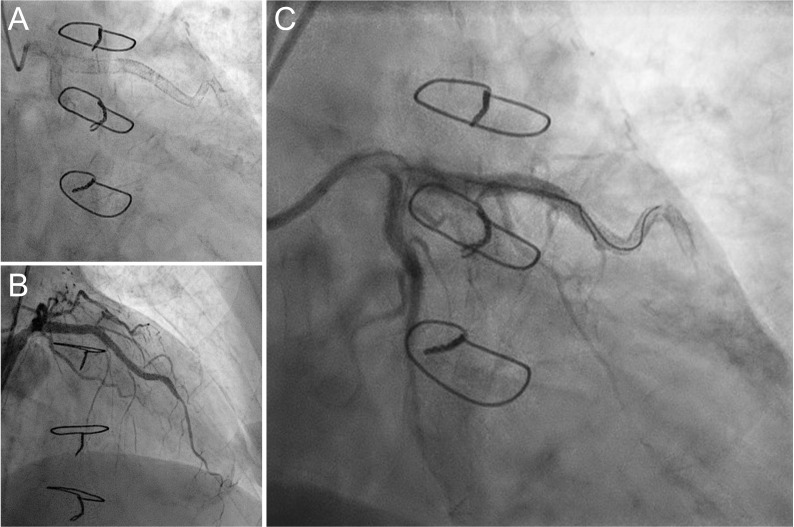
**Coronary angiogram of the left anterior descending artery of patient 2**. A type-IV-SF in the inserting region of the LIMA-ad-LAD-bypass (A) that had previously been treated with implantation of 2 Promus Premier EES and had led to unstable angina pectoris was repaired by deployment of 2 Resolute Integrity ZES (B). However, re-SF occurred only weeks later and further attempts of intervention remained unsuccessful (C).

Patient 2, a 73-year-old male, had undergone coronary artery bypass graft (CABG) surgery with use of the left internal mammary artery (LIMA) more than 20 years prior to the index admission. With progression of the coronary artery disease 3 Promus PREMIER EES had been implanted via the native left main artery (LM) in the left anterior descending artery (LAD) segments 5, 6 and 7. One of the respective EES (3.0 × 16 mm) had been deployed at a high-grade de-novo stenosis of the inserting region of the LIMA-ad-LAD-bypass in segment 7. After a good short-term result, 6 months later an increase of exercise-induced dyspnea had occurred. CAG had again been performed showing ISR of the LIMA-ad-LAD inserting region and a paclitaxel-coated-balloon had been applied (SeQuent Please, B. Braun, Melsungen, Germany) accessing via the LM. 18 months later, the patient had again presented with unstable angina pectoris. CAG had shown recurrent ISR in segment 7 and 1 Promus PREMIER EES (2.5 × 20 mm) had been implanted, again using the LM as access. One month later, the patient had been re-admitted due to angina pectoris. CAG had shown a gap within the previously implanted DES in the insertion region of the LIMA-ad-LAD-bypass, suggesting type-IV-SF (Fig. 2A). Re-PCI and deployment of 2 ZES (2.5 × 18 mm, Resolute Integrity, Medtronic, Minneapolis, MN, USA) had been performed via the LM to stabilize the fracture (Fig. 2B).

At the latest admission the patient presented with NSTEMI. He was transferred to CAG and re-SF of the medial LAD (type IV) with distal TIMI-0-flow and prominent hinge motion was found. Catheterization of the fracture using a BMW guide wire (Abbott, Chicago, IL, USA) through the LM was unsuccessful (Fig. 2C). Further attempts of PCI were not carried out. The patient was commenced with dual antiplatelet therapy.

## 4. Review 

### 4.1 Incidence of Stent Fractures

Stent fractures are common in the field of peripheral vascular interventions and used to be unrecognized in coronary arteries [5]. Chowdhury and Ramos first described a fracture of a coronary BMS in a saphenous vein graft in 2002 [6]. BMS fracture is a rare finding [3, 7, 8], which might be explained by a stabilizing effect of greater neointimal proliferation [9, 10] but also a more difficult diagnosis of fracture due to lower radiopacity [11]. The incidence of SF became more considerable with the introduction of DES. Sianos *et al*. [12] were the first to report SF in DES in two cases involving sirolimus-eluting stents. In their meta-analysis of 8 studies assessing SF, Chakravarty *et al*. [13] report rates of SF ranging between 0.8% and 8.4% with a mean incidence of 4.0%. Notably, all but one SF in their analysis occurred with SES. Similar results were obtained by other studies with an incidence of <1.0% up to 18.6% [10, 11, 14, 15, 16, 17, 18, 19, 20]. An analysis with new generation DES by Schochlow *et al*. [21] showed incidental detection of SF in 8% immediately after implantation and almost 60% in the setting of device failure. The largest multicenter study has been carried out by Kan* et al*. [22] reporting an incidence of SF in 12.3% of the 6555 involved patients, 22.0% of stents and 17.2% of vessels. Limiting to clinical studies however is the varying definition of SF as well as an incomplete angiographic follow-up with the potential to miss out especially on late occurring SF. Accordingly, the highest incidence of SF has been reported in a post-mortem analysis, where Nakazawa *et al*. [23] found evidence of DES fracture in 29% of patients.

### 4.2 Pathomechanism 

The mechanistic culprit behind SF is material fatigue. Biomechanical demands of coronary stents are high. Important factors include vascular biocompatibility, resistance to corrosion, high elasticity and plasticity for expansion, rigidity at body temperature for the maintenance of dilatation and resistance to elastic recoil as well as radiopacity to allow X-ray tracking [24]. While the most broadly used stent backbone material used to be stainless steel, modern 2nd generation DES are preferably fabricated with a cobalt-chromium (Co-Cr) alloy, typically L-605 and MP35N. Radial strength is supported by hoop elements, which are linked by connectors. The latter provide longitudinal stability and their design varies between stent types. In an experimental approach, Ormiston *et al*. [25] showed highest susceptibility to fracture in less flexible stents and stents with three connectors between hoops. Accordingly, most studies have found the relatively inflexible sirolimus-eluting stent (SES) to be the most susceptible to SF [5, 3, 11, 13, 20, 23]. In 10 million cycles of repetitive bending no fracture occurred in the more flexible Element (Boston Scientific, Natick, Massachusetts, MA, USA), Promus (Bostin Scientific, Natick, Massachusetts, MA, USA) and Integrity (Medtronic, Santa Rosa, CA, USA) stents [25]. From their analysis of the American Food and Drug Administration’s (FDA) Manufacturer and User Facility Experience Database (MAUDE) Omar *et al*. [26] also report the highest SF rate in Cypher stents, followed by Xience and Promus stents. But not only the infrastructure of the stent can make a difference. DES are coated with a polymer that controls the release of an antiproliferative drug. Especially in first generation DES, hypersensitivity reactions of the surrounding endothel induced by the polymer with consecutive inflammation has been described. The inflammatory process can then cause late stent malapposition with modification of the mechanical integrity of the device. The results are endothelial hinge points, excessive motion and torsion and finally stent thrombosis or SF [10, 27, 28]. The frequency of polymer-associated inflammation appears to be lower in 2nd generation DES with lowest reported SF rates in everolimus-eluting stents [29].

Further technical improvements have been made to increase flexibility and fracture resistance. Platinum-chromium alloys were recently introduced, allowing for thinner stent struts and higher radiopacity without decreasing radial strength [30, 31, 32, 33]. Kuramitsu *et al*. [34] observed SF in 1.7% of lesions and 2.2% of patients treated with platinum-chromium alloyed everolimus-eluting stents and found a numerically higher incidence of clinically-driven TLR compared to non-SF-lesions. To date, there is no data comparing SF rates in platinum-chromium stents with previous models. However, though not reporting specifically on SF, studies have shown comparable rates of stent thrombosis between cobalt-chromium and platinum-chromium alloy with a significantly lower rate of target lesion failure in platinum-alloyed stents [35]. No stent thrombosis was reported after treatment of small vessels and long lesions, both known as SF-susceptible [36].

A different and promising approach was made with the development of bioresorbable scaffold stents. The latest models, which consist of poly-l-lactic acid (PLLA), degrade over time and were therefore thought to decrease rates of late stent failure. However, bioresorbable PLLA stents have proven inferior compared to non-degradable metallic stent platforms due to increased rates of target lesion restenosis and more frequent target vessel myocardial infarction [37]. Combining the mechanical advantages of non-resorbable metallic platforms and the biodegradability of resorbable stent material bioresorbable metallic stents are currently being advanced. The magnesium-alloyed Magmaris stent (Biotronik AG, Berlin, Germany) has shown safety and efficacy in early registries [38], however randomized controlled trials have not been carried out yet. No data is currently available regarding the use of scaffolds in SF predilection lesions. It is thinkable however, that while the scaffold technology may reduce the risk of fracture in angular and hinge point lesions their rather soft structure might make them prone to breakage or dismantling especially in heavily calcified lesions. Moreover, an essential issue with magnesium-based implants is their rapid degradation and corrosion in aqueous environments like body fluid. A more stable zink-silver (Zn-Ag) alloy has shown promising results in a porcine model with treatment of iliofemoral arteries [39].

Characteristics of the treated lesions are another important factor in the pathomechanism of SF. Numerous studies have shown, that stenting in the RCA is an independent predictor of SF [5, 16, 17, 40, 41, 42, 43, 44]. In their meta-analysis Chakravarty *et al*. [13] estimated 56.4% of SF to occur in the RCA followed by 30.4% in the left anterior descending artery (LAD). SF was least frequent in left main artery lesions [13]. Omar *et al*. [26] reported 47.7% of SF in the RCA, followed by the LAD. The most likely explanation for this clustering of SF events is the tortuosity and contortion of the RCA, leading to higher mechanical force and earlier material fatigue [3, 17, 20]. Ino *et al*. [20] showed a higher degree of hinge motion in the RCA or left circumflex artery (LCX) than in the LAD (31.0° ± 13.1° vs. 22.8° ± 4.9°).

Stenting across angular or hinge regions is a risk factor for SF regardless of the vessel. Park *et al*. [44] found a more than 6-fold higher risk of SF when stenting across an angle of >45°. Popma *et al*. [45], while finding no significant difference in SF incidence between RCA and LAD, reported cyclic angulation changes of 32.3° ± 15.2° in patients with SF. Shaikh *et al*. [11] demonstrated a 14-fold risk increase for SF when stenting across a bend of >75° (Odds Ratio 13.8). Kuramitsu *et al*. [15] reported an Odds Ratio for SF prediction of 14.6 by hinge motion as defined by an at least 16° difference between systole and diastole. And in a study by Park *et al*. [16] 79% of SFs occurred at hinge points, either adjacent to edges of overlapping stents or at angles >45°. But not only does coronary anatomy have an effect on stent mechanics. Vice versa the presence of a stent can significantly alter vascular geometry. Especially stents with low conformability, i.e., small amount of longitudinal flexibility after deployment, can create maldistribution of force during the cardiac cycle predisposing for SF [46].

Other lesional risk factors include ostial [15, 47] and bifurcational lesions [21, 48] as well as plaque calcification [15, 21, 49].

**Fig. 3.**
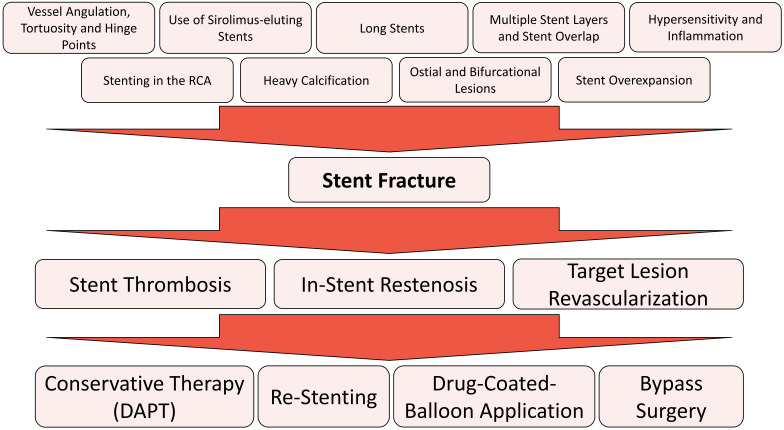
**Risk factors, clinical consequences and therapeutic strategies for stent fracture**. DAPT, dual antiplatelet therapy.

Finally, procedural details can also contribute towards SF. Stent length plays a significant mechanistic role [13]. In the study by Park *et al*. [16], SF occurred in stents with a mean length of 48.3 mm. Omar *et al*. [26] report from the MAUDE database that 65.2% of SF happened in stents longer than 30 mm. Overlapping stents are also a risk factor, since they can change vascular angulation and potentially create new hinge points. Omar *et al*. [26] discuss that half of the SF events in the RCA and LAD registered in the MAUDE database involved lesions with overlapping stents. Stent overlap was also significantly associated with SF in the large meta-analysis by Chakravarty *et al*. [13]. Finally, stent overexpansion can exceed material capacity and induce strut distortion causing early fatigue and SF [5, 12, 21, 50]. Omar *et al*. [26] report a mean postdilation pressure of 18 atm (IQR 16–20) in fractured stents. However, Lee *et al*. [51] showed the occurrence of stent thrombosis in only 0.2% of 1037 patients with intravascular ultrasound (IVUS) guided postdilation with a mean pressure of 18.7 ± 4.1 atm. SF risk from postdilation is likely to vary between different lesions and might be higher in calcified or tortuous lesions [5, 12]. Risk factors of SF are summarized in Fig. 3.

When a stent fractures, the new lesion predisposes for ischemic events. The altered stent geometry, mechanical shear stress and impaired contact of the antiproliferative drug to the endothelium can cause neoproliferation of endothelial tissue and smooth muscle cells, intimal hyperplasia and alteration of hemodynamic factors [13, 15, 52, 53]. Furthermore, complete SF is associated with the formation of coronary aneurysm [10, 45]. As a side note, occasional case reports have been published showing SF as a complication of post-stenting pyogenic infections and mycotic coronary pseudoaneurysm [54, 55]. Furthermore, an infected SF-induced coronary artery aneurysm has been described as a highly probable origin for formation of abscesses and sepsis with *Staphylococcus aureus* [56, 57].

Most SF are reported to occur several months after initial stenting [10, 13]. These events are likely due to the described pathomechanisms and risk factors of material fatigue. However, some SF have been reported only days after stent deployment [5, 50, 58] and are likely to have occurred in high-risk lesions (calcification, angle, overexpansion).

All the above risk factors and mechanisms show, that SF can be seen as a form of “patient-prosthesis mismatch” that requires further improvements in engineering and precision medicine [59].

### 4.3 Diagnosis and Classification

Most studies reporting on SF have based their diagnosis on fluoroscopy [5, 15, 22, 60]. Ino *et al*. [20] discussed, that contrast injection might mask SF lesions and implanted stents should be assessed with and without contrast agents. However, the low spatial resolution of 300 μm limits the diagnostic abilities of fluoroscopy, especially in newer stent models with thin struts. Therefore, additional diagnostic modalities have been introduced, which significantly improved imaging. To enhance visibility of stent struts, high-resolution cine-angiography technologies such as StentBoost (Philips Healthcare, Best, Netherlands) can be helpful [61, 62] and even diagnosed SF in one case, where evidence by intravascular ultrasound (IVUS) was lacking [63]. In a large prospective study by Biscaglia *et al*. [64] enhanced stent visualization techniques proved highly effective and safe in detection of SF during the index PCI. Use of digitally enhanced fluoroscopic imaging can lower the incidence of TLR and MACE [65]. Davlouros *et al*. [66] suggested a staged approach for high-risk patients with routine flat panel digital detector cinefluoroscopy and invasive assessment in case of pathologic initial findings.

IVUS has been used successfully to visualize SF due to its higher spatial resolution (150–200 μm) [5, 10, 13, 15, 19, 67]. Yamada *et al*. [68] reported superiority of IVUS compared to angiography to detect SF.

Optical coherence tomography (OCT) with a spatial resolution of 10–15 μm has seen a rise in use for the differential diagnosis of stent failure [50, 69, 70, 71, 72]. OCT can reproduce the complex spatial stent configuration with high precision and reproducibility [73]. Most recently, Schochlow *et al*. [21] have demonstrated an unexpectedly high prevalence in OCT-diagnosed SF in the setting of elective control examinations.

Since many SF events remain asymptomatic non-invasive diagnostic options are useful. Computer tomography angiography (CTA) has been used as a reliable tool. Hecht *et al*. [48] have reported SF in 28% of patients with ISR as assessed by CTA. They defined evidence of SF with the following criteria: partial or complete (circumferential) gap or “crush” pattern and reduction of Hounsfield units <300 proving the absence of metal. CTA can be superior to fluoroscopic angiography for the detection of SF [74, 75, 76]. However, due to formation of severe artifacts some patients and stents are more suitable for follow-up by CTA than others: Carbone *et al*. [77] suggest patient selection according to stent diameter, stent material and type as well as heart rate and rhythm.

Depending on the diagnostic tool that is used, numerous classifications of SF have been suggested. The first categorizations of SF based on fluoroscopy were done with self-expanding nitinol stents which were implanted in the superficial femoral artery. Scheinert *et al*. [78] described minor SF as single-strut fracture, moderate SF as fracture of more than one strut and severe SF as complete separation of segments. This classification was later adapted for coronary stents by Lee *et al*. [5], Shaikh *et al*. [11] and Kim *et al*. [43]. Allie *et al*. [79] suggested four types of SF with type I being single strut fracture, type II being multiple strut fractures at different sites, type III being complete transverse linear fracture without displacement and type IV being complete stent displacement. Jaff *et al*. [80] introduced an additional type V with formation of a gap between stent fragments. A similar classification was used for coronary stents by Nakazawa* et al*. [23] in their pathological study.

Using IVUS, Doi *et al*. [10] differentiate between partial SF (absence of stent strut across at least one third of the stent) and complete SF (evidence of at least two fragments separated by an image slice with no visible struts).

Schochlow *et al*. [21] reported four OCT-patterns of SF, with pattern 1 being a single stacked strut and pattern 4 being stent transection with or without gap formation.

Finally, Hecht *et al*. [48] distinguished between partial and complete SF using CTA.

### 4.4 Clinical Presentation 

Stent-related adverse events are emerging as a significant issue for the interventional cardiologist. In a recent individual patient pooled study analysis, Madhavan *et al*. [60] showed an incidence of very late stent-related events of 2% per year with all stent types, without an evident plateau over time. SF is one proposed mechanism of stent failure and is known as a major risk factor for ISR, stent thrombosis, TLR and MACE [11, 13, 15, 40, 42, 81, 82, 83]. From their meta-analysis, Chakravarty *et al*. [13] report a significantly higher risk for ISR (38% vs. 8.2%, *p *< 0.001) and TLR (17% vs. 5.6%, *p *< 0.001) in lesions with fractured stents. In the large study by Kan* et al*. [22], SF increased the incidence of ISR, TLR and stent thrombosis more than three-fold. Kashiwagi* et al*. [83] showed a 5-fold higher rate of ISR in SF lesions compared to non-SF lesions. And Ohya *et al*. [82] described a significantly increased risk for clinically driven and all-cause TLR.

Clinical presentation seems to be associated with the extent of SF. SF was seen in 20% of asymptomatic control group devices in a study by Schochlow *et al*. [21], suggesting SF as a common phenomenon often without clinical implications. Lee *et al*. [19] showed that only patients with SF grade III and IV were admitted with acute myocardial infarction. No cardiac deaths occurred in their study. In their post mortem analysis Nakazawa *et al*. [23] found adverse pathologic findings such as thrombosis and restenosis at the SF site in 67% of type V fractures. No significant impact on pathologic findings was seen in type I to type IV SF [23]. While not reporting on the respective grade of SF, Park *et al*. [17] did not find a significant difference in severity of angina pectoris or incidence of acute coronary syndrome in patients with SF compared to a matched control group. And in their large multicenter study, Kan *et al*. [22] did not find a difference in mortality between SF and non-SF patients.

On the other hand, reports of SF leading to STEMI have been published with BMS [7] as well as 1st generation, cobalt-chromium alloyed DES [84, 85]. In a study with 2nd generation DES STEMI occurred in 15.8% of patients with SF [21]. Kuramitsu *et al*. [15] described that the risk for myocardial infarction was more than 12-fold higher in SF compared to non-SF patients. Chhatriwalla *et al*. [86] reported that 12% of patients with SF presented with STEMI or stent thrombosis and 19% with unstable angina or NSTEMI. Omar* et al*. [26] found that one third of patients with SF presented with acute coronary syndrome. Ohya *et al*. [82] report a significantly higher risk for myocardial infarction and very late stent thrombosis in SF patients.

Heterogeneity of the available data might again be due to the varying definition and different diagnostic modalities in SF studies. Undoubtedly, however, SF has a hazardous potential and interventional cardiologists should be familiar with therapeutic strategies to prevent adverse outcome.

### 4.5 Therapeutic Options

Despite the clinical experience with risk factors of SF, management of SF remains challenging and poorly researched. To date, no randomized controlled trials have been carried out to suggest an optimal treatment. Options for management of SF patients are drug therapy, re-stenting, balloon angioplasty and, in some cases, coronary bypass grafting [87]. Omar *et al*. [26] reported from the MAUDE database that half of patients re-admitted with STEMI or stent thrombosis due to SF were treated with DES, 23% with medical therapy alone, 13% with ballon angioplasty and 8% with surgery. While treatment of myocardial infarction due to SF is mainly interventional, there is an ongoing debate how asymptomatic SF lesions should be managed. Since the clinical course of SF, especially minor types with single strut fractures, are often benign and asymptomatic and pose a low risk for adverse cardiac events the cost-benefit ratio of re-intervention is often doubted. Therefore, Adlakha *et al*. [88] proposed to leave asymptomatic SF-related restenosis without treatment and reserve intervention for symptomatic patients. Lee *et al*. [19] advocated treatment of SF with continuation of dual antiplatelet therapy irrespective of symptoms and suggested re-intervention in symptomatic or asymptomatic ISR with >70% stenosis or symptomatic ISR with 50–70% stenosis, which shows positive results in physiological stress test. None of the conservatively treated patients in their study had significantly aggravated restenosis during follow-up nor did cardiac death occur [19]. Ino* et al*. [20] reported similar results with no adverse outcomes in SF patients without significant restenosis who were treated with dual antiplatelet therapy. Park *et al*. [17] only performed re-intervention in SF patients with ISR >70% and achieved excellent results without adverse events. SF Patients without ISR or with acceptable fractional flow reserve were treated conservatively with dual antiplatelet therapy and no patient required TLR during a follow-up period of median 30.5 months [17].

Different strategies are needed for patients presenting with SF and myocardial infarction. Most reports of STEMI due to SF have been treated by re-intervention and stent deployment [7, 84]. However, stenting in mal-apposed stent struts results in a double layer of metal, leading to an increased risk of thrombogenicity, ISR and recurring SF. Case reports have been published using “plain old balloon” [85] or drug-coated balloon (DCB) angioplasty only in a setting of STEMI achieving good short-term results [87]. A balloon only approach to prevent SF in de-novo lesions or stent failure including re-SF could be feasible. The BASKET-SMALL-2-trial has shown non-inferiority of drug-coated balloon (DCB) application compared to DES implantation in small de-novo coronary lesions [89]. DCB was also shown to be non-inferior to 2nd generation DES for treatment of DES ISR [90, 91]. However, specifically in SF DCB application alone could not achieve lower rates of re-ISR and TLR compared to DES implantation [92]. The authors proposed maintained mechanical stress at an SF site as the mechanism of re-re-ISR regardless of the used device [92].

Coronary artery bypass grafting can be seen as the “last resort” in recurrent SF or SF with heavy ISR or stent thrombosis that makes the vessel inaccessible for intervention wires.

## 5. Conclusions

SF is a frequent complication following DES implantation. Minor SF lesions are usually asymptomatic and the risk of ISR, TLR or MACE is low. These lesions can be treated with antiplatelet therapy alone. Major SF however still present a challenge to the interventionalist, as they can be difficult to manage and pose a high risk of re-ISR, re-TLR and adverse cardiac events. Many lesion-specific and procedural risk factors are known that contribute towards material failure and SF. These risk factors are well illustrated by the two cases that we reported above: both patients were stented in areas with sharp angles and marked cyclic motion of the vessels, patient 1 in the RCA (a risk factor itself) and patient 2 at a bypass graft insertion. At least patient 1 was treated with an SES and long stents up to 30 mm were used. And due to recurrent SF and ISR, both patients were provided with multiple stent layers, which amplified mechanical forces and risk of re-SF even further. However, the presentation of both patients with symptomatic acute coronary syndrome has put the interventionalists into a predicament: indeed, in the setting of myocardial infarction efforts of revascularisation are urgently necessary. The risk of recurrent SF due to placement of multiple stent layers was accounted for by use of a Promus PREMIER EES since the platinum chromium platform of Promus Stents was favoured in regard to flexibility compared to cobalt chromium alloys. Further stabilization of the persistent hinge point of the RCA was attempted by implantation of a Resolute Onyx ZES due to putative advantages related to the stent’s construction of a single strand of platinum iridium wire. And in patient 2, after an unsuccessful attempt of DCB application, two Resolute Integrity ZES, consisting of a single cobalt strand, were used to stabilize the hinge point at the insertion of a LIMA-ad-LAD bypass. However, these attempts could not protect the patients from recurrent SF leading to myocardial infarction. With regard to the discussed literature, a balloon-only approach could have been considered a valid alternative strategy in these two patients since it is at least non-inferior to DES implantation.

Since the relevance of coronary interventions will further rise with an ageing population, the number of stent-related complications is also likely to increase. Importantly, elderly patients often combine multiple risk factors for stent failure and SF, such as heavy calcification, vessel tortuosity, presence of vessel grafts, diabetes, renal failure or poor adherence to antiplatelet therapy [93]. Our experience again shows, that once SF has occurred a downward spiral of re-interventions can result. Therefore, the main focus should be on prevention of SF. This can likely be achieved through careful evaluation of the necessity of stent implantation especially in high-risk lesions and consideration of balloon-only strategies if appropriate, adequate lesion preparation by pre-dilation, lithotripsy or application of scoring balloons as well as use of shorter, more flexible stents and avoidance of too aggressive postdilation. Enhanced visualization techniques can be helpful for early SF detection during the index PCI especially in high risk lesions where they can function as a gate-keeper for further IVUS or OCT assessment. If ISR is detected, further assessment by IVUS or OCT should be carried out to elucidate the cause of device failure including SF. And if an SF lesion is verified re-stenting should only be performed after careful consideration of alternative strategies, such as balloon-only or dual antiplatelet therapy.

Future technological developments will hopefully further reduce the incidence of SF and more scientific appreciation of this topic will provide us with evidence based treatment options for these high-risk patients.

## Data Availability

Data and materials are available on request.

## Abbreviations

BMS, Bare metal stent; CABG, Coronary artery bypass graft; CAG, Coronary angiography; DES, Drug-eluting stent; ISR, In-stent restenosis; IVUS, Intravascular ultrasound; LAD, Left anterior descending artery; LCX, Left circumflex artery; LIMA, left internal mammary artery; NSTEMI, Non-ST-segment-elevation myocardial infarction; OCT, Optical coherence tomography; PCI, Percutaneous coronary intervention; RCA, Right coronary artery; SF, Stent Fracture; STEMI, ST-segment-elevation myocardial infarction; TLR, Target-lesion revascularization.
